# Impact of Functional Ingredients on the Technological, Sensory, and Health Properties of Bakery Products

**DOI:** 10.3390/foods13203330

**Published:** 2024-10-20

**Authors:** Roberta Tolve, Barbara Simonato

**Affiliations:** Department of Biotechnology, University of Verona, Strada Le Grazie 15, 37134 Verona, Italy; roberta.tolve@univr.it

Improving well-being, allowing for sustainable lifestyles, and enhancing waste control are aims of the United Nations in the 2030 Agenda for Sustainable Development [1]. These objectives have driven researchers to exploit agro-industrial byproducts as a resource for creating innovative ingredients and fortified foods with enhanced nutritional properties. While agro-food industry waste disposal is a critical environmental issue, recent attainments suggest that the recovery of such byproducts has remarkable economic advantages [2,3].

The emerging popularity of healthy and sustainable diets and the growing consumer demand for functional foods represent recent challenges for investigators and producers. Functional ingredients are represented by different compounds with health-promoting properties that benefit human health [4].

This Special Issue focuses on the various strategies proposed by different authors to recover and re-utilize agro-industrial byproducts that are rich in healthy molecules, such as vitamins, minerals, proteins, and other bioactive compounds involved in modulating humans’ biological functions. These are used to formulate functional bakery goods, creating staple foods that are consumed worldwide.

In the framework of the circular economy and the interest in the recovery and reutilization of agri-food industry byproducts, Pasqualoni et al. developed a low-glycemic bread fortified with red chicory products. There is growing interest in bread formulations with a lower glycemic index (GI). From this perspective, the authors replaced wheat with chickpea flour, various types of resistant starch, and powder from red chicory byproducts (RCP), valuable polyphenols, and dietary fiber sources. Combining RCP with chemically modified tapioca (RS IV) and retrograded starch from tapioca (RS III) allows for the production of bread with a low predicted glycemic index of 52 and 55, respectively, where 55 is the upper value for classifying food as low-pGI [5]. From a technological point of view, the experimental bread had a greater baking loss and increased hardness and chewiness, with decreased cohesiveness, while the volume and specific volume decreased. Incorporating RCP, chickpea flour, and resistant starch from tapioca (RS III and IV) into bread dough is an encouraging approach for developing high-fiber, low-glycemic-index bread, making it an attractive option for health-conscious consumers.

Prasad et al. investigated the inclusion of spinach as agronomic waste to enhance bread’s nutritional value. It has been shown that incorporating spinach produced bread of analogous height but with an altered color and texture compared to the control. As the spinach content increased, total carbohydrates (including starch) decreased, while protein, fiber, and essential molecules such as some vitamins and minerals increased. Additionally, the inclusion of spinach increased bread porosity, while an analysis of the texture showed decreased hardness, chewiness, gumminess, and firmness, and improved cohesiveness. The sensory analysis showed that the taste and odor were less preferred, and general liking reduced with increasing spinach levels, while texture was shown to be acceptable. Nevertheless, the 20% spinach fortification was accepted similarly to the control. Also, in this case, adding a vegetable byproduct to a staple food, such as bread, could be a favorable approach to increasing daily vegetable intake by humans.

Koriyama et al. studied the effects of fortification with the nutrient-rich Moringa oleifera leaf powder (MLP) and roasted MLP (RMLP) on bread characteristics at variable temperatures and times. The authors reported an increase in bread volume with the addition of RMLP treated at 130 °C for ≥20 min. Furthermore, RMLP allowed for regular dough fermentation, while MLP interfered with carbon dioxide production. Therefore, bread fortified with MLP bread generally shows low fermentation and crumb expansion, but these effects are decreased by heat treatment. These results emphasize the effect of heat treatment in diminishing the influences of MLP on bread fermentation and swelling.

Guan et al. evaluated the effect of tannic acid (TA) as a dough additive on enhancing bread quality. TA supplementation at 0.1% and 0.3% concentrations significantly improved bread’s antioxidant activity and enhanced its texture by decreasing crumb hardness, gumminess, and chewiness, and increasing the specific volume. Moreover, adding TA did not modify the crust color but increased the bread’s porosity. Additionally, the study demonstrated no negative impact on sensory properties, suggesting TA’s commercial viability as a dough improver.

As reported by Cerdá-Bernad et al., saffron (*Crocus sativus* L.) has many byproducts in the production of the most expensive spice in the world. To obtain 1 kg of the spice, about 350 kg of tepals are discarded. Responding to this challenge, some authors aimed to study the nutritional, physicochemical, and sensory properties of wheat and spelt bread supplemented with saffron floral byproducts in a 0, 2.5%, 5%, and 10% (*w*/*w*) ratio. The antioxidant compounds during *in vitro* digestion were also evaluated. The results demonstrated improved dietary fiber and mineral content in the 10% saffron floral byproduct- fortified bread. 

Moreover, the increments in phenolic content and antioxidant ability were stable throughout the *in vitro* digestion processes. Finally, the addition of saffron flowers altered the organoleptic properties of bread. Thus, saffron floral byproducts represent sustainable ingredients that can be used to design new functional foods.

Ozón et al., focusing on sustainability, developed another approach, fortifying bread with chia seed byproducts: bioactive peptides from industrial oilseeds. Wheat bread was supplemented with chia expeller hydrolysate flour. ThisClick or tap here to enter text. fortified bread achieved good overall consumer liking according to an affective test. The peptides obtained from the protein hydrolysis of the chia expeller demonstrated technological properties and chemical compositions that are suitable for developing bakery products.

This method provides a novel, eco-friendly option for recovering nutrients from agro-industrial byproducts, obtaining bread with antioxidant properties due to the functional peptides derived from the chia expeller. 

Bianchi et al. developed an alternative approach, reducing muffin fat content by using wine lees (WL), a cheap byproduct from wine production, as a fat substitute. The results showed that by substituting sunflower oil with WL, the batter consistency of the fat-replaced muffins was lower than the control, while the hardness and chewiness were higher. Muffins with 25% and 50% fat substitutions had a better volume, increased fiber content, and decreased calorie content.

The food industry has recently explored the use of other types of fats to substitute traditional solid ones [6]. Oleogels containing hydroxypropyl methylcellulose (HPMC), xanthan gum (XG), and olive oil replaced shortening in white pan bread preparation to reduce the saturated fat and trans-fat. The study conducted by Kim et al. displayed that a 50% shortening replacement had no impact on pan bread volume compared to the control and reduced bread retrogradation.

This provides a practical solution to decrease harmful fats in bread through using oleogels as healthier alternative fats.

The rise in gluten-related disorders has driven the demand for gluten-free (GF) products by consumers. Koh et al. evaluated the effect of adding different kinds of spent tea leaf powder (STLP) from green, oolong, and black teas at 8% *w*/*w* in GF shortbread cookies. The addition of STLP to cookies increased moisture, total dietary fiber (soluble and insoluble), and ash contents, but decreased carbohydrate and energy value compared to control cookies. Adding STLP improved the cookies’ antioxidant properties and the samples’ shelf-life quality. The moisture content, water activity, texture, and microbiology were retained. Thus, STLP represents a valuable byproduct and could be a promising ingredient in the formulation of gluten-free goods with improved nutritional, technological, and sensory properties. 

Baek et al. assessed the use of O-free wheat, a lower-allergy grain in ω-gliadins with a reduced amount of glutenins and gliadins, to formulate cookies.

The authors showed that the experimental cookies were like the ones prepared with flour with low gluten content, which is suitable for preparing these baked goods. This research suggests adopting O-free flour in developing allergy-friendly cookies.

## Conclusions

In conclusion, this Special Issue showcases the diverse approaches and innovations in the utilization of functional ingredients from agro-industrial byproducts to improve bakery products’ nutritional, sensory, and technological properties. The research highlights not only the potential to enhance health benefits, such as lowering the glycemic index and increasing fiber content and antioxidant capacities, but also the ability to repurpose waste into valuable components of food products in line with sustainability goals. From fortifying bread with resistant starch, spinach, and Moringa to incorporating chia peptides and wine lees in muffins, each case presents a compelling argument for eco-friendly solutions to food production. The studies also underscore the importance of consumer acceptance in developing these functional foods, as sensory attributes play a crucial role in their success. Ultimately, these studies align with global efforts towards developing sustainable food systems, reducing waste, and promoting healthier diets, contributing significantly to the objectives of the United Nations 2030 Agenda for Sustainable Development.
